# Health of the City

**Published:** 1848-01

**Authors:** 


### HEALTH OF THE CITY.

Typhoid fever seems to be establishing itself in our city as one of our indigenous diseases. Within a few days we have heard of several cases, one of which, in a young man aged about 21 years, had a fatal termination.

